# Response to comment on 'The distribution of antibiotic use and its association with antibiotic resistance'

**DOI:** 10.7554/eLife.47124

**Published:** 2019-05-03

**Authors:** Scott W Olesen, Marc Lipsitch, Yonatan H Grad

**Affiliations:** 1 Department of Immunology and Infectious DiseasesHarvard TH Chan School of Public HealthBostonUnited States; 2 Department of EpidemiologyHarvard TH Chan School of Public HealthBostonUnited States; 3 Center for Communicable Disease DynamicsHarvard TH Chan School of Public HealthBostonUnited States; 4 Department of Medicine, Division of Infectious DiseasesBrigham and Women's Hospital, Harvard Medical SchoolBostonUnited States; Imperial College LondonUnited Kingdom; Imperial College LondonUnited Kingdom

**Keywords:** antibiotic resistance, antibiotic prescribing, reverse causality, None

## Abstract

We are writing to reply to the comment by Pouwels et al., 2019 about our recent study (Olesen et al., 2018) on antibiotic use and antibiotic resistance.

## Introduction

We thank Pouwels and colleagues for their thoughtful critique and appreciate their concern for the role that analyses like ours could play in public health policy (Pouwels et al., 2019). Moreover, we appreciate their willingness to discuss and refine these ideas through both formal and informal conversations, as we work together towards the shared goal of addressing the challenges posed by antimicrobial resistance. Although we disagree on certain points, discussed below, we note that our disagreements depend on remarkably subtle arguments about specific aspects of prescriber behavior and its role in reverse causality. We take this as an overall sign that research into the epidemiology of antibiotic resistance will benefit from further study of individual prescriber behavior in response to patient-level risks and population-level prevalence of antibiotic resistance.

In our original analysis, we evaluated several questions about the relationships between antibiotic use and resistance. First, we described the distribution of antibiotic use across the population and showed that it is uneven, as some individuals had no antibiotic pharmacy claims and others had many. Second, we characterized the landscape of correlations between antibiotic use and resistance for antibiotics and many pathogens. Third, we investigated whether first or repeat antibiotic use is more strongly associated with resistance at the population level. To do so, we partitioned a person's number of antibiotic pharmacy claims in a year into the sum of ‘first use’ – zero if the person had no claims for that antibiotic, one if they had at least one claim – and ‘repeat use’, the number of claims beyond the first. Previous reports speculated that repeat use would have a greater effect on population-level antibiotic resistance than first use, in other words, that ‘intensive’ use of antibiotics in a few people is more important for resistance than ‘extensive’ use across many people. In our analysis, we found no evidence that repeat use was more strongly associated with resistance. If true, these results imply that antibiotic stewardship should not necessarily focus their attention on a few, high-consuming antibiotic users.

## Discussion

Pouwels et al., 2019 raised several concerns with the results about the relationship between population-level antibiotic resistance and first vs repeat antibiotic use. First, they argue that reverse causality (rational prescriber response to local resistance rates) dominates over forward causality (the biological selection for resistance) in associations between antibiotic use and resistance, so that our analytical results about the relationships between first use, repeat use, and antibiotic resistance do not support our conclusions about how antibiotic stewardship should be performed. Second, they argue that the relative roles of forward and reverse causality differ for first and repeat use in a way that further undermines our original conclusions. Third, they argue that analyzing use of all antibiotics, rather than use of particular classes of antibiotics, changes the relative role of forward and reverse causality and find evidence that, if this assumption were correct, would even further undermine our conclusions. Finally, they argue that there are many confounders, data problems, and open methodological questions that limit the applicability of our results for determining public health policy.

First, does reverse causality (rational prescriber response to local resistance rates) dominate over forward causality (the biological selection for resistance) in associations between antibiotic use and resistance, as Pouwels et al., 2019 maintain? We disagree. As outpatient prescribers do not in general rationally account for resistance rates (Simpson et al., 2007), we are not convinced that reverse causality dominates. However, the degree to which clinicians alter their antibiotic prescribing practice in response to local rates of antibiotic resistance is an empirical question, and our debate could be resolved by better direct quantification of reverse causality.

Second, does reverse causality influence repeat use more than it influences first use? We again disagree with Pouwels et al., 2019. We expect that high population-level resistance against an antibiotic would discourage rational prescribers from both first and repeat use of that antibiotic. In contrast, in cases of low population-level resistance, rational prescribers would be more discouraged against repeat use than first use, because resistance may arise because of antibiotic exposure in that patient. In other words, prior antibiotic use is a more important risk factor for patient-level resistance when population-level resistance is low compared to when population-level resistance is high. We therefore expect that reverse causality, when population-level resistance affects individual-level prescribing, would have a greater influence on first use than repeat use. If this is true, then the associations between first use and resistance we observed in our original analyses, which tended to be positive, are actually underestimates of the forward-causal effect of antibiotic use on resistance, in fact strengthening the interpretation of our original result that first use has a more important forward-causal effect on resistance than repeat use.

Third, how does analyzing overall antibiotic, rather than the use of particular antibiotic classes, affect the interpretation of the resulting models? We disagree with the interpretation of this kind of analysis in Pouwels et al., 2019. In the original analyses (Olesen et al., 2018), we found that the associations between repeat use of an antibiotic and resistance to that antibiotic tend to be negative. Re-analyzing our data, Pouwels et al., 2019 found that associations between overall repeat antibiotic use and resistance to particular classes of antibiotics tend to be positive. They asserted that focusing on overall antibiotic use limits the role of reverse causality and concluded that repeat use has a more important forward-causality effect on resistance than repeat use, the opposite of our original finding. Although we generally agree with their logic about how focusing on overall use limits the role of reverse causality, we expect that focusing on overall use limits the role of forward causality as well. It is not clear to us that reverse causality is limited to a greater degree than is forward causality. If this is true, the finding of Pouwels et al., 2019 – that overall repeat use is better associated with resistance than overall first use – does not contradict our original finding – that first use of individual antibiotic classes is better associated with resistance to those antibiotics than repeat use of those antibiotics. We explain our logic in the context of first and repeat use separately.

With respect to first use, Pouwels et al., 2019 asserted that overall first use – that is, the proportion of people who claimed any antibiotic – is an indicator of how many patients require an antibiotic prescription. This measure should be less affected by antibiotic resistance levels than first use rates for any particular class of antibiotics. Thus, models of overall first use rather than first use of particular antibiotic classes should be less susceptible to reverse causality. Although we agree with this logic about reverse causality, it does not address how the change in model predictors affects forward causality. In the absence of reverse causality, use of antibiotic A should be a better predictor for resistance to A than use of any antibiotic, even given co-selection and other mechanisms by which use of other antibiotics can select for resistance to antibiotic A. In other words, using overall first use as a predictor dilutes forward causality. The relative importance of the two effects is not clear to us: is the dilution of forward causality sufficiently counterbalanced by the diminution of reverse causality?

We similarly question the logic that models of overall repeat use rather than repeat use of particular antibiotic classes should be less susceptible to reverse causality. Pouwels et al., 2019 mentioned that a high resistance rate to a first-line therapy will mean that, on average, more unique antibiotics will be required to cure the infection. Overall repeat use captures this drug-switching, emphasizing reverse causality: when resistance to an antibiotic is high for a given pathogen, more antibiotic prescriptions, especially for antibiotics of different classes, will be used to treat infections due to that pathogen. As with first use, it is unclear to us why overall repeat antibiotic use should have a stronger forward-causal, biologically selective effect on resistance to a particular class of antibiotics, compared to repeat use of antibiotics in that particular class. Again, however, the lack of strong evidence about the role of antibiotic resistance in prescribers' decisions to switch antibiotic treatments for a patient limits a productive debate.

In contrast to the above, we heartily agree with the final point of Pouwels et al., 2019 that there are many confounders, data problems, and open methodological questions that may limit the applicability of our results for determining public health policy. We welcome further research in the areas of reverse causality and the determination of precisely which antibiotic use, by whom and of what drugs, are the principal drivers of resistance. We also applaud efforts to generate more reliable data sources so that estimates of antibiotic resistance in the US need not rely on, as we did, a convenience samples of hospital antibiotic resistance reports. However, in the meantime, we hold that today's public health policy must proceed based on the best available evidence.
